# Predicting reading ability in teenagers who are deaf or hard of hearing: A longitudinal analysis of language and reading

**DOI:** 10.1016/j.ridd.2018.04.007

**Published:** 2018-06

**Authors:** Sarah Worsfold, Merle Mahon, Hannah Pimperton, Jim Stevenson, Colin Kennedy

**Affiliations:** a Faculty of Medicine, University of Southampton, Southampton, SO16 6YD, UK; b Language and Cognition Research Department, UCL, London, WC1X 8EE, UK; c Psychology, Faculty of Social and Human Sciences, University of Southampton, Southampton, SO17 1BJ, UK; d University Hospital Southampton NHS Foundation Trust, UK

**Keywords:** Permanent childhood hearing loss, Reading accuracy, Reading comprehension, Expressive language, Language comprehension, Longitudinal design

## Abstract

**Background:**

Deaf and hard of hearing (D/HH) children and young people are known to show group-level deficits in spoken language and reading abilities relative to their hearing peers. However, there is little evidence on the longitudinal predictive relationships between language and reading in this population.

**Aims:**

To determine the extent to which differences in spoken language ability in childhood predict reading ability in D/HH adolescents.

**Methods:**

and procedures: Participants were drawn from a population-based cohort study and comprised 53 D/HH teenagers, who used spoken language, and a comparison group of 38 normally hearing teenagers. All had completed standardised measures of spoken language (expression and comprehension) and reading (accuracy and comprehension) at 6–10 and 13–19 years of age.

**Outcomes:**

and results: Forced entry stepwise regression showed that, after taking reading ability at age 8 years into account, language scores at age 8 years did not add significantly to the prediction of Reading Accuracy z-scores at age 17 years (change in R^2^ = 0.01, p = .459) but did make a significant contribution to the prediction of Reading Comprehension z-scores at age 17 years (change in R^2^  = 0.17, p < .001).

**Conclusions:**

and implications: In D/HH individuals who are spoken language users, expressive and receptive language skills in middle childhood predict reading comprehension ability in adolescence. Continued intervention to support language development beyond primary school has the potential to benefit reading comprehension and hence educational access for D/HH adolescents.

## What this paper adds?

The difficulties D/HH children have in acquiring reading skills have been repeatedly demonstrated but longitudinal studies of D/HH children identifying aspects of underlying language skills that contribute to variation in reading skills are rare. The present study examines the stability of reading skills from middle childhood to adolescence in D/HH individuals and shows moderate stability in Reading Comprehension and a high level of stability in Reading Accuracy scores. It also shows that variation in the language ability of D/HH children in middle childhood is predictive of their Reading Comprehension in late adolescence. This predictive relationship is over and above the continuity in Reading Comprehension between childhood and late adolescence. The same was not found for Reading Accuracy; language ability in middle childhood was not a significant predictor of adolescent word reading skills. Within the D/HH group, differences in the relationship between severity of hearing loss and reading ability in late adolescence were accounted for by differences in language ability. The results of this study contribute to the case for continued targeted intervention on language skills for D/HH individuals beyond primary school. Maximising their language ability, and consequently their ability to use reading to access learning, is likely to enhance their educational attainment and subsequent life chances

## 1 Introduction

Despite recent technological improvements, such as digital hearing aids and cochlear implants, and earlier diagnosis and management of babies born deaf or hard of hearing (D/HH), reading ability in D/HH children and young people continues to lag behind that of their hearing peers (Wauters, van Bon, & Tellings, 2006; Moeller, Tomblin, Yoshinaga-Itano, Connor, & Jerger, 2007; Harris & Terlektsi, 2011; Qi & Mitchell, 2011; Pimperton et al., 2016; Harris, Terlektsi, & Kyle, 2017a). Moreover, with increasing age a widening gap in reading achievement between D/HH and hearing children has been observed (Blair, Peterson, & Viehwg, 1985; Marschark & Harris, 1996; Kyle and Harris, 2010, Kyle and Harris, 2011). As children get older, reading takes on an increasingly important role in enabling them to access the curriculum; they move from ‘learning to read’ to ‘reading to learn’. Thus the reading deficits shown by the D/HH population are likely to have an increasingly significant impact on their educational attainment and subsequent employment opportunities. The continuing importance of reading ability and educational attainment for the later occupational status of D/HH individuals in adulthood has been shown by Walter and Dirmyer (2013).

Despite group-level deficits in their reading ability, D/HH children and young people show substantial individual variation in reading skills, with some reading at an age-appropriate level (Kyle & Harris, 2006, 2010Kyle and Harris, 2010; Harris and Terlektsi, 2011; Pimperton et al., 2016). The question of what drives this variation is an important one because identifying these drivers may raise potential avenues for intervention to support reading development in this group.

In hearing children, the causal contribution of underlying language abilities to reading development has been repeatedly demonstrated through good quality longitudinal and intervention studies (see Hulme & Snowling, 2014, for review). Phonological language skills (e.g. phonological awareness, letter-sound knowledge) appear to be most important for reading accuracy (i.e. decoding written words into their phonological form) (Muter, Hulme, Snowling, & Stevenson, 2004; National Institute for Literacy, 2008; Bowyer-Crane et al., 2008; Caravolas et al., 2012) whereas non-phonological broader oral language skills (e.g. vocabulary, grammatical knowledge, morphological skills) appear to be most important for reading comprehension (i.e. understanding the meaning of what is read) (Nation, Cocksey, Taylor, & Bishop, 2010; Clarke, Snowling, Truelove, & Hulme, 2010; Fricke, Bowyer-Crane, Haley, Hulme, & Snowling, 2013).

The phonological and non-phonological language skills identified as playing a causal role in reading development in hearing children are all skills which D/HH children find difficult to acquire and in which they show deficits relative to the skills of hearing peers (Moeller et al., 2007; Musselman, 2000; Wake, Hughes, Poulakis, Collins, & Rickards, 2004). Taken together this suggests that variation in reading skills of D/HH children may be driven by variation in these underlying language skills. Consistent with this, factors related to audiological experience, such as severity of hearing loss, age at identification and age at cochlear implantation, that influence language development in D/HH children, have also been found to relate to their reading development (Moeller et al., 2007; Archbold et al., 2008; McCann et al., 2009).

The findings outlined above imply a similar role for language skills in causally driving the development of reading skills in D/HH children, as in hearing children. However, there is a dearth of good quality longitudinal and intervention studies to test whether, and if so which, language skills play a causal role in the reading development of D/HH children.

The most extensive set of studies of the role of language in reading for D/HH individuals is on phonological coding and awareness (PCA). Mayberry, del Giudice, and Lieberman (2011) identified 25 studies that had examined PCA abilities and their relationship with reading abilities in deaf children and adults. The assessment of reading proficiency was based on some studies that measured reading comprehension and others reading accuracy. They estimated that 11% of the variance in reading proficiency was explained by PCA, similar to the 12% of variance that was thus explained in a meta-analysis of studies with hearing participants (Bus and van IJzendoorn, 1999). Around half of studies included in the Mayberry et al. (2011) meta-analysis showed evidence of an effect of PCA on reading proficiency in the deaf participants, while the other half did not. It is likely that differences between studies in terms of the format of the PCA tasks used (e.g. auditory input-oral response vs. visual input-nonverbal response), aspects of reading assessed (accuracy vs. comprehension), and populations included (e.g. children vs. adults, oral language vs. sign language users) will have contributed to the lack of consistency in results.

Vocabulary and grammatical knowledge are key broader language skills that have been causally associated with reading comprehension development in hearing readers and have also been shown to relate to reading skills in deaf children (Geers, 2003; Barajas, Gonzalez-Cuenca, & Carrero, 2016). In line with this, the Mayberry et al. (2011) meta-analysis also reported that a broader measure of general language ability (signed or spoken) explained 35% of the variance in reading proficiency in those studies that included such a language measure, and was the factor with the strongest relationship to reading. They concluded that “deaf readers, like hearing readers, are more likely to become successful readers when they bring a strong language foundation to the reading process” (Mayberry et al., 2011, p.181).

In commenting on the Mayberry et al. (2011) meta-analysis, Kyle, Campbell, and MacSweeney (2016) point out that the majority of the studies on language and reading in deaf children are based on correlational studies assessing the concurrent associations between these two aspects of development. They argue that longitudinal studies would provide a more robust test of the nature of these possible causal relationships. Kyle and Harris, 2010, Kyle and Harris, 2011 have reported on two such studies of D/HH children in middle childhood. They found that vocabulary knowledge at mean age 7 years 10 months predicted reading comprehension outcomes one year later (Kyle & Harris, 2010). The same significant longitudinal relationship was found between vocabulary knowledge at 8 years 10 months and reading comprehension at 10 years 11 months. This finding supports an association between vocabulary knowledge in the development of reading comprehension. They also showed that word reading (i.e. reading accuracy) at this age was significantly predicted, albeit to a lesser extent, by vocabulary knowledge and that neither reading comprehension nor word reading was predicted by phonological awareness. Similar findings on the predictive role of vocabulary knowledge on reading accuracy were found in the second longitudinal study in a sample of D/HH children tested at mean age 5 years 8 months, 6 years 8 months and 7 years 11 months (Kyle & Harris, 2011). An important element of both these studies was the demonstration that vocabulary was associated with later reading performance even after adjusting for earlier reading performance. In other words, it controlled for the fact that the strongest predictor of reading will be the same ability measured at an earlier time point, known as the ‘auto-regressive effect’.

To summarise, there is strong evidence in hearing children that phonological language skills predict word reading accuracy and non-phonological broader language skills predict reading comprehension (Hulme & Snowling, 2014). The evidence base is weaker for D/HH children, with the majority of evidence coming from cross-sectional correlation studies, but there is some longitudinal evidence for the role of vocabulary knowledge development of reading comprehension and reading accuracy for these children too (Kyle and Harris, 2010, Kyle and Harris, 2011). More longitudinal studies are needed to contribute to this evidence base and to clarify the nature of the relationships between language skills and reading proficiency in D/HH children and young people. This has not previously been addressed in older D/HH children and adolescents, in whom relationships identified between reading and language in the early stages of literacy acquisition may no longer apply.

This paper reports on an analysis of relationships between language and reading measured on the first occasion at approximately 8 years and on the second occasion at approximately 17 years of age in a population-based sample of D/HH children with bilateral moderate-profound permanent childhood hearing loss (PCHL) who used spoken English as their primary form of communication. This sample was drawn from a prospective cohort study that addressed the impact of Universal Newborn Hearing Screening (UNHS) on early confirmation of hearing loss (Kennedy et al., 1998; Kennedy, McCann, Campbell, Kimm & Thornton, 2005) and subsequent language and reading outcomes (Kennedy et al., 2006; McCann et al., 2009; Pimperton et al., 2016; Pimperton et al., 2017).

The main question addressed in this paper is whether expressive language, receptive vocabulary and grammatical skills in middle childhood predict reading accuracy and reading comprehension abilities in adolescence, after adjusting for the effects of auto-regressive continuities in reading. The longitudinal design of the study also makes it uniquely well-placed to provide novel evidence on the stability of reading accuracy and reading comprehension scores between middle childhood and adolescence for D/HH individuals.

## 2 Methods

### 2.1 Participants

The D/HH and hearing participants were all drawn from a 1992-97 birth cohort of 157,000 children born in eight districts of southern England (see Kennedy et al., 2006). Of 168 children in the birth cohort with PCHL, 160 were contactable and 120 gave consent to be included in the study. These 120 children in the D/HH group had been diagnosed with bilateral PCHL > 40 dB hearing level (dB HL) in the better ear which was not known to be post-natally acquired. Severity of hearing loss was categorised as moderate (40–69 dB HL), severe (70–94 dB HL) or profound (≥95 dB HL) according to four-frequency averaging of the better ear pure-tone thresholds at 0.5, 1, 2 and 4 kHz. Maternal education was classified according to the 2001 UK census. The hearing comparison group (HCG; N = 63) was randomly selected from babies born on the same day and in the same district as participants in the D/HH group.

Seventy-six of the 120 D/HH and 38 of the 63 HCG participated at Time 2 (T2). Seventeen of the 76 D/HH participants did not complete the spoken language assessments at T2. This was either because they used British Sign Language (BSL) as their preferred language, rendering these spoken English assessments inappropriate, or because they had severe additional disabilities that precluded the development of sufficient language to attempt the tests.

The analyses presented in this paper are based on the 53 participants with PCHL and 38 participants in the HCG for whom data were available on tests of reading and language at both T1 (mean age 8.0 years) and also at T2 (mean age 17.3 years). The requirement for reading and language measures to be available at both time points was necessary to allow all the longitudinal analyses to be conducted on the same sample of children. If reading and language measures were available, participants were not excluded because of the presence of additional disabilities. The two such participants both had learning disabilities i.e. nonverbal IQ < 70 at T1 (see 3.4 for results of sensitivity analysis).

The demographic characteristics of the samples are presented in Table 1. Sample attrition over the approximately eight years between these two assessment time points, coupled with inclusion only of those participants who were spoken language users, reduced the sample size for the longitudinal analysis presented here. The annual rate of attrition was 4% since their assessment at primary school. This degree of attrition is relatively low for follow-up studies of long-term paediatric conditions (Karlson & Rapoff, 2009). Attrition was principally due to the participants not responding to requests to participate in later phases of the study (for details see Pimperton et al., 2017, Fig. 1). The summary statistics presented in Table 1 show that both the PCHL and HCG groups in the present study were similar in their demographic characteristics to those of the initial samples.

**Table 1:** Demographic characteristics of teenage study participants. ^a^The ‘initial sample’ was a population-based sample of children with PCHL and a normally hearing comparison group that had previously participated in a study of language and reading at primary school age at mean age 8.0 years. ^b^O-level examinations (now replaced by general certificates of education) are usually taken at 16 years of age.

	PCHL group		Hearing comparison group	
	Initial sample^a^	Teenage sample	Initial sample^a^	Teenage sample
	n = 120	n = 53	n = 63	n = 38
Mean age (SD) in years at Time 1 assessment	7.87 (1.3)	7.99 (1.1)	8.12 (1.0)	8.1 (1.1)
Mean age (SD) in years at Time 2 assessment	n/a	17.2 (1.4)	n/a	16.3 (1.2)
Female sex *n* (%)	53 (44)	26 (49)	26 (41)	13 (34)
Severity of hearing loss *n* (%)				
Moderate	62 (52)	29 (55)	n/a	n/a
Severe	29 (24)	11 (21)		
Profound	29 (24)	13 (24)		
English as main language at home *n* (%)	99 (83)	48 (91)	60 (95)	36 (95)
Cochlear Implant	16 (13)	10 (19)	n/a	n/a
Maternal education				
‘no quals or <5 O-levels^b^	43 (36)	15 (28)	25 (40)	11 (29)
>5 O-levels or some A-levels^c^	62 (52)	30 (57)	25 (40)	16 (42)
University or higher degree	14 (12)	8 (15)	13 (21)	11 (29)

PCHL = Bilateral permanent childhood hearing impairment > 40 dB HL.

^a^The ‘initial sample’ was a population-based sample of children with PCHL and a normally hearing comparison group that had previously participated in a study of language and reading at primary school age at mean age 8.0 years.

^b^O-level examinations (now replaced by general certificates of education) are usually taken at 16 years of age.

^c^A-level examinations (now replaced by A2s) are taken two years later as qualifications for entry to higher education.

### 2.2 Procedure

The study was approved by the Southampton and South West Hamphsire Research Ethics Committee. Written informed consent for participation in the study was obtained from principal caregivers at T1and T2 and from the teenage participants at T2.

At both T1 and T2, each participant’s reading, language and non-verbal ability (N-VA) was assessed in a quiet room at home or school by a trained researcher. At the same time we collected, from participants and their families, information on characteristics including maternal education level and languages used in the home. The most recently available audiological data were taken from audiology and cochlear implant centre records – for participants with hearing aids from the last annual review and for those with cochlear implants, unaided pure-tone thresholds obtained during the original implant assessment.

### 2.3 Materials

The group mean score and standard-deviation scores in the HCG were used to derive z scores for the D/HH participants. The z-score for a D/HH participant is equal to the number of standard deviations of the distribution of scores in the HCG participants by which the D/HH participant’s age-adjusted score differs from the mean score of the HCG participants.

#### 2.3.1 Reading

Reading Accuracy and Reading Comprehension at T1 were measured using the Wechsler Objective Reading Dimensions (WORD) (Wechsler, 1993) and at T2 using the newly available York Assessment of Reading for Comprehension Secondary Edition (YARC), (Stothard, Hulme, Clarke, Barmby, & Snowling, 2010). The YARC was used at T2 because, with minor adaptations approved by the test designers (see Pimperton et al., 2016), we could use this measure with both spoken and sign language users in our sample as it did not involve reading aloud. The difficulty of reading comprehension tasks undertaken in the YARC was determined by word reading performance rather than chronological age, which was also felt to be more appropriate for our D/HH sample.

#### 2.3.2 Language skills

##### 2.3.2.1 Language comprehension

At both T1 and T2 the Test for Reception of Grammar (TROG-2; Bishop, 2003) and The British Picture Vocabulary Scale (BPVS-3; Dunn, Dunn, & National Foundation for Education Research, 2009), were used to assess receptive skills for spoken English grammar and vocabulary respectively. TROG-2 contains test items that assess understanding of increasingly complex grammatical contrasts, including plurals, passives, negatives, and relative clauses. In both tests participants point to a picture from a choice of four alternatives that corresponds to a spoken stimulus. The z scores from the TROG-2 and the BPVS were highly correlated at both Time 1 (n = 53, r = 0.82) and Time 2 (n = 53, r = 0.67). Accordingly the two z-scores were averaged to produce Language Comprehension scores at Time 1 and at Time 2.

##### 2.3.2.2 Expressive language

Scores from age appropriate narrative assessments (Renfrew Bus Story Test (Renfrew 1995) at T1, and Expression, Reception and the Recall of Narrative Instrument (ERRNI; Bishop, 2004) at T2) provided an Expressive Language score.

The Renfrew Bus Story Test was developed for 3–8 year olds and involved children listening to a story told by the researcher supported by a series of pictures that correspond to the story. They then retold the story using the pictures as prompts and their retelling was audio-recorded and transcribed by the administering researcher. Two z scores from this measure (amount of information in the narrative and the average length of utterances i.e. number of words used) were averaged into an Expressive Language composite score (see Kennedy et al., 2006 for details). In a reliability exercise, data for 15 randomly chosen participants were independently transcribed by a second rater. No discrepancy of word content was found between transcriptions but the point at which a sentence should pause (e.g. commas, full stops) was a subjective decision. The Bland-Altman method (Bland & Altman, 1986) was used for assessing agreement between the two transcripts on average length of the longest 5 sentences and the information score. There was an average difference of 1.1 units between the two transcripts on these two scores (0.43 and 1.53 respectively) and variability of these differences was acceptable (95% confidence intervals −1.80 to 2.65 and −5.68 to 8.75 respectively).

At T2 the ERRNI was selected, as it was similar in design to the Bus Story Test but suitable for use with adolescents. It requires test-takers to produce a narrative based on a series of cartoon pictures then reproduce that narrative, though this time without the support of pictures. As with the Bus Story Test, the ERRNI narratives were audio-recorded and transcribed by the researcher who had taken the recording. The ERRNI produced three scores: an Initial score for the quality of participants’ initial narratives, a Recall score for the quality of their recalled narrative, and a Mean Length of Utterance (MLU) score which gave the average length of utterances across both the initial and recalled narratives. The Initial and the Recall z scores were highly correlated (n = 53, r = 0.72) and therefore averaged to form an Expressive Language-Information score at T2. The MLU measure was less highly correlated with the other two T2 Expressive Language measures (n = 53, r = 0.20 and 0.32) and was therefore analysed separately.

Following Whitehouse, Line, Watt, & Bishop (2009), an inter-rater reliability exercise was carried out to check the reliability of the ERRNI scoring. A random sample of 12 narratives (12% of the total) was transcribed and scored by a second rater. There was good agreement between the two ratings for all three scores (intraclass r: Initial = 0.82; Recall = 0.90; MLU = 0.95).

##### 2.3.2.3 Speech intelligibility

Parents’ assessment of connected speech intelligibility was assessed at T1 and T2 using the Speech Intelligibility Rating Scale (Allen, Nikolopoulos, & O'Donoghue, 1998). This 5 point scale gives short descriptors of each level with 1 being ‘completely unintelligible’ and 5 being ‘intelligible to all listeners’ and was developed for use in cochlear implant assessment.

#### 2.3.3 Non-verbal ability

At T1 we assessed Non-verbal Ability using the Raven’s Standard Progressive Matrices (Styles, Raven, & Raven, 1998). At T2 the 20 min timed version (Hamel & Schmittman, 2006) was used. Participants were given twenty minutes to work their way through a series of progressively more complex matrix reasoning puzzles. Raw scores reflecting the total number of correct items out of a possible 60 were calculated.

#### 2.3.4 Data analysis

Regression analyses were conducted using SPSS 24 (IBM Corp., 2016). Following the recommendations by Kraemer and Blasey (2004), all the independent measures were centred before being included in the regression analyses.

For this longitudinal analysis it was important to compare directly the same D/HH participants relative to the same NH control group at both time points (T1 and T2). The language z scores at T1, which had initially been derived relative to the scores for all participants in the HCG at T1, were therefore recalculated relative to the scores of only those HCG participants at T1 that also participated at T2. These recalculated z scores were used in the subsequent analyses.

A preliminary test was conducted to determine whether there were possible covariates that might be confounding predictors of reading at T2. As recommended by Kraemer (2015) the number of putative covariates was kept to a minimum by requiring them to be significantly correlated with either of the T2 reading measures. Forced entry was used to enter the independent variables in blocks with the T1 reading score entering at Step 1, the covariates entering in Step 2 followed by the T1 Expressive Language and Language Comprehension measures at Step 3. The R^2^ change from step 2 was used to test for any significant effects of T1 language on T2 reading. Only the R^2^ values and their associated F tests are reported, as these are unaffected by collinearity.

For each regression, the distribution of residuals was tested for skewness and kurtosis and the Kolmogorov-Smirnov test of normality was applied. For the prediction of T2 Reading Accuracy and for T2 Reading Comprehension these tests all had p values greater than .05 indicating that the distribution of the residuals did not deviate significantly from normal.

Some previous studies on reading development in the D/HH have excluded children with low non-verbal cognitive abilities (Kyle & Harris, 2011). Accordingly, a sensitivity analysis was conducted to determine whether their inclusion had an impact on the results in the present study.

## 3 Results

### 3.1 Comparison of the PCHL and HCG mean scores

The Reading Accuracy, Reading Comprehension, Expressive Language, Language Comprehension and Non-verbal Ability scores at T1 and T2 for the PCHL group and the HCG are compared in Table 2. The scores for the HCG (n = 38) on all measures are mean = 0.00 and SD = 1. Scores of participants with PCHL were lower than those of the HCG on all language and reading measures with the exception of T2 Expressive Language-Information score and T2 Expressive Language-MLU score. There was a wider range of achievement at T2 than T1 in the DH/H group and the spread of the D/HH group reading scores was larger than that in the HCG with approximately 25% achieving reading scores above the HCG mean at T2. The spread of D/HH group reading scores had also increased by age 17 years and was larger than that in the HCG. At T1 those with PCHL had significantly lower Non-verbal Ability scores than the HCG. There is a degree of normalisation over time of Non-verbal Ability in the DH/H group and consequently there is no significant difference from the HCG at T2. The increase in Non-verbal Ability in the D/HH group was large enough to be clinically important and was also statistically significant (SMD = −0.60, 95%CI −0.98 to −0.21, t = 4.41, df = 52, p < .001).

**Table 2:** Group mean reading, language and non-verbal ability z-scores for participants with PCHL and difference from z-scores of the HCG.

	PCHL group z-scores (n = 53)		Standardised mean difference between PCHL group and HCG z-scores^a^ (n = 38)			
	Group mean	SD	Standardised mean difference (95% CI)	t	df	p
**Time 1**						
Reading Accuracy	−0.89	1.07	−0.85 (−1.29 to −0.42)	4.03	89	≪.001
Reading Comprehension	−0.87	1.18	−0.78 (−1.22 to −0.35)	3.70	89	≪.001
Expressive Language	−1.11	1.53	−0.83 (−1.26 to −0.40)	4.28	86.42	≪.001
Language Comprehension	−1.95	1.49	−1.49 (−1.95 to −1.02)	7.29	86.38	≪.001
Non-verbal Ability	−0.69	0.91	−0.72 (−1.15 to −0.30)	3.40	89	≪.001
**Time 2**						
Reading Accuracy	−1.33	1.55	−0.99 (−1.42 to −0.54)	4.96	88.6	≪.001
Reading Comprehension	−1.02	1.43	−0.80 (−1.24 to −0.37)	3.81	88.97	≪.001
Expressive Language-Info	−0.19	1.11	−0.18 (−0.60 to 0.24)	0.86	89	.390
Expressive Language-MLU	−0.15	0.92	−0.16 (−0.57 to 0.26)	0.76	89	.449
Language Comprehension	−1.76	2.30	−0.94 (−1.38 to −0.50)	5.13	69.65	≪.001
Non-verbal Ability	−0.17	0.82	−0.19 (−0.60 to 0.22)	0.90	89	.388

PCHL = bilateral permanent childhood hearing loss > 40 dB HL; HCG = hearing comparison group; Info = Information; MLU = mean length of utterance.

a The z-scores for the HCG (n = 38) on all measures are mean = 0.00 and SD = 1. The standardised mean difference is Cohen’s d (difference in means divided by pooled standard deviation).

At T2 all those in the D/HH group were intelligible to listeners who had at least some experience of the speech of children with PCHL (Appendix A).

### 3.2 Testing for potentially confounding covariates

Correlations between potential covariates and T2 Reading Accuracy and T2 Reading Comprehension for PCHL (n = 53) were calculated (see Table 3). It should be noted that the Reading Accuracy and Reading Comprehension scores were age-adjusted z-scores and consequently age would not be a confounder. Gender was not related to either T2 Reading Accuracy or T2 Reading Comprehension and therefore was not included as a covariate. All the other covariates were significantly correlated with at least one T2 reading score and were retained. These covariates were mother’s education, first language English, severity of hearing loss and T1 Non-verbal Ability.

**Table 3:** Correlations between Reading Accuracy and Reading Comprehension in PCHL group at Time 2 and potential covariates.

	Time 2 Reading Accuracy (n = 53)	Time 2 Reading Comprehension (n = 53)
	*r*	*r*
English first language^a^	−0.29*	−0.16
Mother’s education^b^	.29*	0.21
Gender^c^	−0.02	0.08
Severity of hearing loss^d^	−0.32*	−0.34*
Time 1 Non-verbal Ability	.29*	0.14

PCHL = bilateral permanent childhood hearing loss > 40 dB HL.

* p ≪ 0.05.

a Binary variable coded +0.5 = yes, −0.5 = no.

b Binary variable coded −0.5 = less than A levels, +0.5 = A level or higher.

c Binary coded variable −0.5 = male, +0.5 = female.

d Coded −1 = moderate, 0 = severe, +1 = profound.

### 3.3 Predicting Time 2 scores

The results of forced entry stepwise regression predicting Reading Accuracy at T2 are shown in Table 4. There was a high level of stability in the Reading Accuracy scores (Step 1 R^2^ = 0.63, p < .001). This indicates that 63% of the variance in T2 Reading Accuracy scores at mean age 17 years was predicted by Reading Accuracy scores measured some 9 years earlier. The covariates jointly added 0.07 to the R^2^ (p = .04). The T1 language scores did not add significantly to the prediction of the T2 Reading Accuracy scores (Step 3 change in R^2^ = 0.01, p = .459). A regression analysis adding T1 Expressive Language and Language Comprehension sequentially (i.e. step 3a then step 3b) showed that neither explained significant unique variance in T2 Reading Accuracy i.e. there was no significant change in R^2^ at step 3b whichever order they were entered.

**Table 4:** Forced entry stepwise regression predicting Reading Accuracy at Time 2 in PCHL group (n = 53).

	R^2^	R^2^ Change	F	df	*p*
Step 1	0.63	0.63	89.42	1.51	<.001
Time 1 Reading Accuracy					
Step 2					
English first language, Mother’s education, Severity of hearing loss, Time 1 Non-verbal Ability	0.70	0.07	2.67	4.47	.04
Step 3	0.71	0.01	0.79	2.45	.459
Time 1 Expressive Language					
Time 1 Language Comprehension					

PCHL = bilateral permanent childhood hearing loss > 40 dB HL.

Forced entry stepwise regression predicting Reading Comprehension scores at T2 showed that there was a moderate degree of stability in the Reading Comprehension scores (Step 1 R^2^ = 0.43, p < .001) i.e. reading comprehension scores at T1 accounted for 43% of the variance of scores of the same skill at T2 (Table 5). The covariates did not add significantly to the prediction of the T2 Reading Comprehension (Step 2 change in R^2^ = 0.04). However, the two T1 language scores made a significant contribution to the prediction of T2 Reading Comprehension (Step 3 change in R^2 ^= 0.17, p < .001). A regression analysis adding T1 Expressive Language and Language Comprehension sequentially (i.e. step 3a then step 3b) showed that although there was much shared variance, each of the language variables also made a significant unique contribution to the explained variance. Language Comprehension explained a significant additional 6% (p < .001) of the variance in Reading Comprehension beyond that explained by Expressive Language. Expressive Language accounted for an additional 3% (p < .05) of the variance in Reading Comprehension beyond that explained by Language Comprehension.

**Table 5:** Forced entry stepwise regression predicting Reading Comprehension scores at Time 2 in PCHL group.

	R^2^	R^2^ Change	F	df	*p*
Step 1	0.43	0.43	37.91	1.51	<.001
Time 1 Reading Comprehension					
Step 2					
English first language, Mother’s education, Severity of hearing loss, Time 1 Non-verbal Ability	0.46	0.04	0.83	4.47	.51
Step 3	0.63	.17	10.03	2.45	<.001
Time 1 Expressive Language,					
Time 1 Language Comprehension					

PCHL = bilateral permanent childhood hearing loss > 40 dB HL.

### 3.4 Sensitivity test for other disabilities

The analyses reported in 3.1–3.3 were repeated with the exclusion of the two participants with learning disabilities i.e. non-verbal IQ < 70. No substantive changes were shown in the results in terms of the parameter estimates and their associated p values.

## 4 Discussion

This study examined relationships between language and reading, measured in middle childhood and adolescence, in a population-based sample of D/HH children with bilateral PCHL > 40 dB using spoken English as their chief mode of communication. Approximately half of the sample had a moderate hearing loss and the majority had language skills within 2 SDs of their HCG peers. The D/HH participants had significantly lower scores than the HCG on reading measures in both middle childhood and adolescence. Such group-level deficits in reading replicate findings previously reported in children with hearing loss of varying severities (Moeller et al., 2007; Geers & Hayes, 2011; Harris & Terlektsi, 2011). In the present study at mean age 17 years, the reading scores of the D/HH were approximately 1 SD below those of the HCG and this gap was slightly greater than it had been for the same participants at mean age 8 years. A similar pattern of relative decline in reading ability was reported in 11 year olds by Kyle & Harris (2010), where children who were severe-profoundly D/HH made only 0.3 of a grade improvement in reading age per year between the ages of 8 and 11 years. Early superiority in reading skills may have enabled the HCG to read more demanding material more frequently than their D/HH peers, increasing the skill gap and resulting in a rich-get-richer ‘Matthew effect’ (Stanovich, 1986) which is likely to impact on their educational outcomes and subsequent life chances.

When examining the stability of reading scores in D/HH individuals in this study, we found, as expected, that the majority of variance of reading scores at T2 was accounted for by scores of the same abilities measured at T1. This extends the demonstration in typically developing young people with normal hearing that early literacy was highly predictive of reading ability at 17 years of age (Cunningham & Stanovich, 1997). In that study this effect was mediated, in part, by the impact of early reading on exposure to print. For some D/HH individuals such a virtuous circle of early reading enhancing later achievement may be more difficult to initiate, given the difficulties they can experience in acquiring reading skills.

The main focus of this paper was to determine the extent to which language abilities contribute to later reading abilities in D/HH children. We found that receptive and expressive spoken language abilities in middle childhood accounted for significant variance in adolescent Reading Comprehension but not Reading Accuracy. A key aspect of this result is that the analyses adjusted for Reading Accuracy or Comprehension performance in middle childhood when predicting the same skill in adolescence thus demonstrating that these language variables explain variance in reading comprehension development from middle childhood to adolescence. In addition, we found that Expressive Language and Language Comprehension measured at age 8 years each explained small but significant unique portions of the variance in Reading Comprehension at age 17 years in addition to the variance they explained in common.

The finding that broader oral language skills predicted reading comprehension outcomes in this sample of D/HH adolescents is consistent with findings in hearing and in younger deaf children regarding the salience of these skills for predicting reading comprehension development. (e.g. Nation et al., 2010; Kyle & Harris, 2010). This consistency with the findings of Kyle and Harris (2010) is observed despite the differing distribution of hearing loss severity within the two samples. Taken together these consistent findings suggest a common role for broader language skills in facilitating reading comprehension for hearing and for D/HH children across the severity spectrum.

Conversely the lack of predictive relationship between these same language skills and reading accuracy is consistent with findings from hearing children that reading accuracy is best predicted by measures of phonological skills (e.g. Muter et al., 2004). However, a significant limitation of our study was the absence of a measure of phonological ability at age 8 years, which meant we were unable to assess the predictive relationships between phonological language skills and reading accuracy and comprehension outcomes in this sample. Kyle and Harris, 2010, Kyle and Harris, 2011 found that receptive vocabulary is an important predictor of word reading in younger D/HH children. T1 vocabulary might have predicted T2 reading accuracy in our study, but as receptive vocabulary and receptive grammar scores were combined to create a composite receptive language score (see Methods) and vocabulary was not examined separately we are not able to say. The fact that receptive language was not an important determinant of reading scores in the present study may be due to differences in the ages or the distribution of hearing loss severity and communication modes between the two study populations.

The receptive language tests selected for this study have been widely used with deaf children in research, clinical and educational settings. Although standardised on hearing children they require a non-verbal response which avoided wrongly scoring mis-pronounced responses as if they were the consequence of limited syntax or vocabulary ability. Furthermore the tests had been standardised on a wide age range allowing use of same measures at both time points.

For the expressive language tests, the Bus Story (T1) requires high levels of oral language comprehension (the participant listens to then retells the story). However, the ERRNI does not (participants generate the story themselves from pictures). We have previously highlighted this difference (Pimperton et al., 2017) as a potential contributor to the reduction in the expressive language deficit of the D/HH group relative to the hearing group at T2. Improvement in speech intelligibility of the D/HH group from T1 to T2 (see additional Appendix A) is another potential contributor to this effect. This likely involvement of language comprehension in the expressive language measure at T1 is consistent with the finding that there is much shared variance between T1 language comprehension and T1 expressive language when predicting T2 reading comprehension; though T1 expressive language still predicts a small but significant amount of unique variance beyond that explained by T1 language comprehension.

An unexpected finding was improvement in non-verbal ability in those with PCHL between the ages of 8 and 17 years. This does not appear to have been reported in previous studies. The finding is given weight by being obtained in a longitudinally studied sample where the same children provided non-verbal ability scores at the two ages. However, the finding may be sample specific and would benefit from replication in other samples e.g. the sample studied by Wake et al. (2004). Alternatively, it may relate to having sufficient language to support reasoning processes but we know of no evidence of such a mechanism accounting for this relative normalisation of non-verbal ability.

There are consistent reports that severity of hearing loss is related to reading development in D/HH individuals (e.g. Moeller, Tomblin, & the OCHL Collbaration, 2015). What has been less studied is what might mediate this effect. It is unlikely that factors linked to the aetiology of the hearing loss also acted to influence reading development, except those that affected non-verbal ability (which was included in our analysis) or were associated with progressive hearing loss. In the present study, severity of hearing loss was related to reading score but the severity of hearing loss per se did not predict reading ability once the effects of language ability (using our composite expressive + receptive language score) were controlled. This suggests that the association between severity of hearing loss and the difficulties in acquiring reading may be mediated by the effect of hearing loss on language ability.

This study has a number of strengths including the longitudinal design and the population-based sampling. In such a population based longitudinal study over a 9-year period there is inevitably sample attrition. In the present study the evidence suggests that in most respects the retained participants were representative of the original sample.

As the teenagers assessed in this study were born in 1992–1997, the question arises whether the reading skills of D/HH children born more recently might show different levels of reading, and different relationships between language and reading skills, as a result of advances in hearing device technology and in early identification and intervention since the mid 1990s. A recent report of reading skills of children with severe-profound PCHL aged 5–7 years when recruited in 2013–14 (i.e. born in 2006–09) described little improvement in phonological awareness or reading ability compared with a similar study sample born 10 years earlier, but did report improvements in vocabulary levels (Harris, Terlektsi & Kyle, 2017b). Predictive relationships between the language and reading variables were consistent across the two cohorts in some cases (e.g. vocabulary) but differed in others (e.g. phonological awareness). A replication of the longitudinal analyses reported in the current study in cohorts born more recently would be valuable to address whether the pattern of findings reported here generalises to these later-born cohorts.

Our findings provide support for the value of intervention to foster language development in D/HH children (Rees et al., 2015; Gilliver, Cupples, Ching, Leigh, & Gunnourie, 2016; Richels et al., 2016) but importantly suggest that continued support for broader oral language skills into the secondary school years could continue to be an effective way to enhance reading comprehension ability in this population. Maximising the reading comprehension skills in D/HH individuals of secondary school age is vital because of the increasing demands on comprehension skills as text complexity increases in the secondary school years, and the vital role that reading comprehension plays in broader educational success at secondary school.

## 5 Conclusions and implications

This study extends our understanding of reading development in D/HH children and adolescents by highlighting the predictive relationships between language skills in middle childhood and reading comprehension in adolescence, even when adjusting for earlier reading skills. These findings contribute to the case for continued targeted intervention on language skills for D/HH individuals beyond primary school. Maximising their language ability, and consequently their ability to use reading to access learning, is likely to enhance their educational attainment and subsequent life chances.

## Conflicts of interest

The authors declare that they have no conflicts of interest in presenting the results in this paper.

## Figures and Tables

**Table:** 

Speech Intelligibility Rating Scale ratings	Frequency	Per cent
**Time 1**		
Unintelligible	1	1.9
Intelligible to those with experience	7	13.2
Intelligible to those with little experience	24	45.3
Intelligible to all	21	39.6
**Time 2**		
Unintelligible	0	0
Intelligible to those with experience	4	7.5
Intelligible to those with little experience	19	37.3
Intelligible to all	28	54.9

## Appendix A

Parent ratings on Speech Intelligibility Rating Scale in PCHL group

PCHL = bilateral permanent childhood hearing loss > 40 dB HL
